# Disease-specific plasma levels of mitokines FGF21, GDF15, and Humanin in type II diabetes and Alzheimer’s disease in comparison with healthy aging

**DOI:** 10.1007/s11357-020-00287-w

**Published:** 2020-10-31

**Authors:** Maria Conte, Jacopo Sabbatinelli, Antonio Chiariello, Morena Martucci, Aurelia Santoro, Daniela Monti, Marina Arcaro, Daniela Galimberti, Elio Scarpini, Anna Rita Bonfigli, Angelica Giuliani, Fabiola Olivieri, Claudio Franceschi, Stefano Salvioli

**Affiliations:** 1 Department of Experimental, Diagnostic and Specialty Medicine (DIMES), University of Bologna, Bologna, Italy; 2 Interdepartmental Center “Alma Mater Research Institute on Global Challenges and Climate Change (Alma Climate)”, University of Bologna, Bologna, Italy; 3 Department of Clinical and Molecular Sciences (DISCLIMO), Università Politecnica delle Marche, Ancona, Italy; 4 Department of Experimental and Clinical Biomedical Sciences “Mario Serio”, University of Florence, Florence, Italy; 5 Fondazione Ca’ Granda IRCCS Ospedale Maggiore Policlinico, Milan, Italy; 6 Dino Ferrari Center, University of Milan, Milan, Italy; 7 Scientific Direction, IRCCS INRCA, Ancona, Italy; 8 Center of Clinical Pathology and Innovative Therapy, IRCCS INRCA, Ancona, Italy; 9 Laboratory of Systems Medicine of Healthy Aging and Department of Applied Mathematics, Lobachevsky University, Nizhny Novgorod, Russia

**Keywords:** GDF15, FGF21, Humanin, Aging, AD, T2D

## Abstract

Fibroblast Growth Factor 21 (FGF21), Growth Differentiation Factor 15 (GDF15), and Humanin (HN) are mitochondrial stress-related mitokines, whose role in health and disease is still debated. In this study, we confirmed that their plasma levels are positively correlated with age in healthy subjects. However, when looking at patients with type 2 diabetes (T2D) or Alzheimer’s disease (AD), two age-related diseases sharing a mitochondrial impairment, we found that GDF15 is elevated in T2D but not in AD and represents a risk factor for T2D complications, while FGF21 and HN are lower in AD but not in T2D. Moreover, FGF21 reaches the highest levels in centenarian’ offspring, a model of successful aging. As a whole, these data indicate that (i) the adaptive mitokine response observed in healthy aging is lost in age-related diseases, (ii) a common expression pattern of mitokines does not emerge in T2D and AD, suggesting an unpredicted complexity and disease-specificity, and (iii) FGF21 emerges as a candidate marker of healthy aging.

**Supplementary Information:**

The online version of this article (10.1007/s11357-020-00287-w) contains supplementary material, which is available to authorized users.

## Introduction

Aging is a complex and progressive phenomenon characterized by a decline and a reshape in normal biological functions, leading to the appearance of the aging phenotype, which is usually accompanied by the presence of chronic degenerative diseases, even though a minority of cases can escape this destiny and reach extreme age in good health. We have conceptualized that healthy aging, geriatric syndromes (such as frailty), and age-related diseases (ARDs) are part of a *continuum* where precise boundaries do not exist but the rate of aging can be different, leading to longevity (low rate) or ARDs (high rate) [1]. In the last decades, several theories have been proposed about the molecular and cellular mechanisms at the basis of the aging process and it is generally acknowledged that the mitochondria play a key role. Mitochondria are fundamental to produce cellular energy currency and are involved in a variety of metabolic pathways. The aging process is characterized by a progressive mitochondrial dysfunction accompanied by increased production of Reactive Oxygen Species (ROS), considered as one of the key hallmarks of a variety of several age-related pathologies, such as neurodegenerative diseases, metabolic diseases, cardiovascular diseases, and cancer [2, 3]. Mitochondrial dysfunction represents a stress condition that elicits an adaptive response which is not confined into the cell but can also spread to distal tissues by means of soluble mediators indicated as mitokines [4]. Mitokines include a variety of circulating proteins and peptides released by different cell types that act as hormones [5, 6]. Fibroblast Growth Factor 21 (FGF21), Growth Differentiation Factor 15 (GDF15), and Humanin (HN) are among the most studied mitokines. Consistently with the idea that mitochondrial dysfunction increases with age, the plasma levels of these mitokines are strongly associated with aging and many ARDs [5–7].

Alzheimer’s diseases (AD) and type 2 diabetes (T2D) are among the most common and important ARDs, and both share a metabolic and inflammatory background [7, 8]. Accordingly, AD has been also proposed as Type 3 diabetes [9]. Most importantly, AD and T2D also share a mitochondrial dysfunction [10, 11], suggesting a possible similarity in the expression of mitokines in the two conditions. FGF21 is a stress hormone belonging to the FGF family. There is evidence that FGF21 counteracts age-related metabolic changes and promotes the maintenance of health and longevity. Studies on animal models demonstrate that the overexpression of FGF21 is associated to the extension of lifespan, and thus, FGF21 is considered as a pro-longevity hormone [12, 13] with anti-inflammatory activity [14, 15]. It was also demonstrated that FGF21 is implicated in the regulation of energy metabolism and homeostasis [16], and circulating FGF21 levels have been reported to be increased in metabolic stress conditions, such as obesity, insulin resistance, and T2D, [13, 17] and interpreted as a sort of homeostatic response to counteract the metabolic stress. Accordingly, FGF21 is in fact considered as a possible therapeutic approach for metabolic disorders such as T2D [18, 19]. Other studies also suggest a neuroprotective role against pathologies such as AD [20, 21].

GDF15 is a stress response molecule belonging to the transforming growth factor-β (TGF-β) superfamily. GDF15 is produced in response to mitochondrial and inflammatory stressors and is involved in many ARDs, such as cancer, T2D, obesity, and cardiovascular and neurodegenerative diseases. Recent studies demonstrate that GDF15 strongly correlates with aging and is considered a marker of biological age [5, 22–24]. Moreover, GDF15 seems to have protective roles against local and systemic inflammation [25, 26]. It is now well known that high GDF15 levels are associated with insulin resistance and T2D, and GDF15 has been therefore considered a diagnostic biomarker of T2D [27, 28]. However, little is known about its possible involvement in AD.

HN is a 24-amino acid mitochondrial DNA-encoded peptide involved in many biological processes associated with inflammatory response, oxidative stress, and apoptosis [29, 30]. HN was discovered for its neuroprotective role against AD [31]; however, HN acts as cytoprotective molecule also in T2D, cardiovascular disease, atherosclerosis, and cancer [6]. The role of HN in aging is still debated. Some studies showed a decrease of HN with aging [32, 33], while others showed an age-related increase of HN at plasma level [5, 34].

Within the framework of the *continuum* hypothesis mentioned earlier, we aimed to test the hypothesis that it is possible to identify a sort of trend for mitokine expression from healthy aging to ARDs, as well as similarities and differences between ARDs sharing a common ground of mitochondrial dysfunction such as T2D and AD. To this purpose, we have studied the plasma levels of these three mitokines in a group of > 500 age-matched elderly characterized by different types of aging: healthy controls, centenarians’ offspring (OFF), and patients affected by T2D or AD. Of note, OFF are characterized by a better health status with respect to their age-matched peers and are considered as a reliable example of healthy aging [35, 36]. We found that mitokine patterns are different not only between healthy people (OFF and controls) and patients but also within patient groups, suggesting that mitokine expression regulation is more complex than expected.

## Material and methods

### Subjects

A total of 569 subjects in the age range 52–88 years were recruited and divided into five groups, according to their healthy or pathological status: 102 centenarian offspring (OFF), 92 healthy controls (HC), 162 type 2 diabetes (T2D) patients without complications (T2DnC) and 93 T2D patients with complications (T2DC), and 120 patients with Alzheimer’s disease (AD) (Table 1). All subjects were enrolled in Italy in the framework of previous projects as described in Bucci et al. [36] for OFF, Testa et al. [37] for T2D patients and HC, and Sims et al. [38] for AD patients. The study protocols were approved by the following Ethical Committees (EC): EC of Sant’Orsola-Malpighi University Hospital, Bologna, Italy (Ethical clearance EM 157/2011/U issued on Nov. 25, 2011) for OFF, Institutional Review Board of Italian National Research Center on Aging (INRCA) for HC and T2D (Ethical clearance 34/CdB/03), and Comitato Etico Milano Area 2 for AD. All subjects signed informed consent before blood withdrawal and interviews to collect data on health status, clinical anamnesis, and details on medications. Subjects affected by malignant neoplasia and/or those in therapy with immune suppressor drugs (like cyclosporine, methotrexate, glucocorticoids) or anticoagulant drugs were excluded from the study. As far health status, OFF and HC were free of clinically evident major diseases. For T2D patients, the inclusion criteria, the clinical information collected from each subject, and the presence of diabetic complications were as reported in Testa et al. and Mensà et al. [37, 39]. All AD patients were sporadic cases, and no one had T2D-related comorbidities. No difference in terms of mitokine levels was observed between early onset and late onset patients, so they were considered together.

**Table 1:** Study samples

	OFF	HC	T2DnC	T2DC	AD
No. of subjects	102	92	162	93	120
Age range (mean ± SD)	54–88 years (71.10 ± 7.67)	60–87 years (68.76 ± 6.18)	60–81 years (68.46 ± 5.30)	60–87 years (69.34 ± 5.29)	52–87 years (72.02 ± 7.97)
Sex (*N*)	68 F, 34 M	45 F, 47 M	87 F, 75 M	42 F, 51 M	65 F, 55 M

*OFF* centenarians’ offspring, *HC* healthy controls, *T2DnC* T2D patients without complications, *T2DC* T2D patients with complications, *AD* Alzheimer’s disease patients

### Data collection

For all subjects, blood was drawn in the morning after overnight fasting. All samples were processed to collect plasma. Plasma was obtained within 4 h from venipuncture by centrifugation at 2000*g* for 20 min at 4 °C, rapidly frozen and stored at − 80 °C.

Serum concentrations of HbA1c, uric acid, azotemia, triglycerides, ApoA1, ApoB, and highly sensitive C-reactive protein were measured by standard biochemical assays in HC and T2D patients. Estimated glomerular filtration rate (eGFR) was calculated according to CKD-EPI (Chronic Kidney Disease Epidemiology Collaboration) equation based on serum creatinine, age, sex, and ethnicity [40].

APOE genotyping was performed by 7500 Fast Real Time PCR System (Applied Biosystems): DNA samples were genotyped for two APOE single-nucleotide polymorphisms (SNPs; rs429358 and rs7412) and relative results defined APOE ε2, ε3, and ε4 alleles. We assigned APOE ε4 status as APOE ε4 negative (ε4−) for APOE ε2/ε3 and APOE ε3/ε3 (non-carriers) and APOE ε4 positive (ε4+) for APOE ε2/ε4, APOE ε3/ε4, or APOE ε4/ε4 (carriers of at least one copy of the APOE ε4 allele).

GDF15, FGF21, and HN concentrations were determined in plasma samples by ELISA assay using commercial kits, highly specific for the detection of each human mitokine: R&D for GDF15 (DGD150: intra- and inter-assay coefficient of variation (CV) range: 10.9–1.1% and 4.1–3.0%, respectively; minimum detectable dose 2.0 pg/mL) and FGF21 (DF2100: intra- and inter-assay CV range: 10.2–3.0% and 10.6–3.1%, respectively; minimum detectable dose 4.67 pg/mL) and CUSABIO for HN (CSB-EL015084HU: intra- and inter-assay CV range: 5.5–0.7% and 11.8–3.4%, respectively; minimum detectable dose 7 pg/mL), according to the manufacturer’s instructions. In all the samples, GDF15, FGF21, and HN were measured in duplicate, and the mean values were used in the statistical analyses. The standard curves were determined by simultaneously analyzing a dilution series of standard samples. The final data were obtained in a blind set up by the operator. Synergy™ fluorometer (Bio-Tek Instruments, Winooski, Vermont, USA) was used to read the absorbance of each plates.

### Statistical analysis

The data were analyzed with non-parametric tests since they did not follow a normal distribution. In particular, the comparisons among OFF, HC, T2D, and AD patients were performed by using Kruskal-Wallis test, while the comparison between ApoE4+ and ApoE4− AD patients was performed by Mann-Whitney test. The Bonferroni correction was applied. The relationships between each mitokine levels and age were calculated by Spearman rank correlation test and regression analysis. A regression analysis was performed for HC and T2D to evaluate the relationship between BMI and FGF21. The difference between the two regression curves was estimated by the following linear model: *y* = m + FGF21 + Group_i_ + FGF21xGroup_i_, where *y* = BMI values, FGF21 = covariate effect of FGF21, and Group_i_ = fixed effect of the *i*th group (HC, T2D). Mediation analysis was performed using model 4 of the PROCESS Macro for SPSS with a bootstrapping procedure involving 10,000 re-samples to generate model estimates and confidence intervals.

A multinomial logistic regression model using the enter method was constructed to identify factors associated with the presence of T2D. Model fit was assessed using the Hosmer-Lemeshow goodness-of-fit test. The proportion of variance explained by the final model was determined using the Nagelkerke *R*^2^ statistic.

Analysis of covariance (ANCOVA) followed by post-hoc tests for multiple comparisons was used to compare the mean differences in mitokine levels after adjustment for age and sex, and, in case of T2D, glucose-lowering treatment (see the “Results” section).

Receiver operating characteristics (ROC) curves were constructed to assess the discriminatory ability of mitokines in T2D complications. Youden’s index was used to calculate the best cut-off values, where appropriate. Multiple ROC curves were compared using the DeLong method [41] (DeLong et al. 1988).

Significance was accepted as *p* < 0.05. Data are expressed as mean ± SE or SD. All data were analyzed using the SPSS 23.0 for Windows software (SPSS Inc.; Chicago, IL, USA).

## Results

### The plasma levels of mitokines are disease-specific

We measured the levels of GDF15, FGF21, and HN in 569 plasma samples from T2D patients, with (T2DC) or without (T2DnC) complications, AD patients, healthy age-matched controls (HC), and centenarians’ offspring (OFF), see Table 1. A comparison of the plasma levels of each mitokine among all groups was performed. In agreement with our previous data [5], no gender difference was found (data not shown); therefore, males and females were pooled together.

**Fig. 1:**
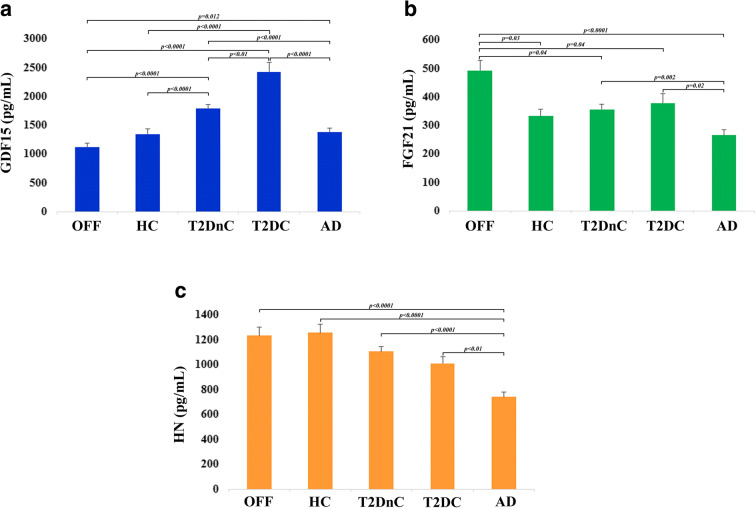
Plasma levels of GDF15, FGF21 and HN in healthy and pathological conditions. Circulating plasma levels of GDF15 (**a**), FGF21 (**b**), and HN (**c**) in centenarians’ offspring (OFF), healthy controls (HC), T2D patients without complications (T2DnC) and T2D patients with complications (T2DC), and Alzheimer’s disease patients (AD). Data are expressed as mean ± SE. *p* values were determined by Kruskal-Wallis test with Bonferroni correction

GDF15 levels were significantly higher in T2D patients as a whole when compared to HC, OFF and AD patients (*p* < 0.0001). Moreover, within T2D patients, T2DC showed higher levels with respect to T2DnC (*p* < 0.01). As far as AD patients, their levels of GDF15 were slightly but significantly higher with respect to OFF (*p* = 0.012), but not to HC (Fig. 1a). This last result suggests that AD is not associated with dramatic alterations of GDF15 concentrations at systemic level.

A different trend was observed for FGF21. In this case, OFF showed the highest levels of FGF21 as compared to all the other groups. No difference was found between HC and T2D or AD patients; however, a slight but significant difference was found between T2D and AD patients (T2DnC vs AD *p* = 0.002, and T2DC vs AD *p* = 0.02) (Fig. 1b). These results suggest that elevated levels of FGF21 are associated with health, while low levels are more likely to be present in people with neurodegenerative problems.

Plasma levels of HN were significantly lower in AD patients with respect to all the other groups. No significant difference between healthy subjects (OFF and HC) and T2D patients was observed (Fig. 1c).

Taken together, these data suggest that these mitokines are modulated in a disease-specific way.

**Fig. 2:**
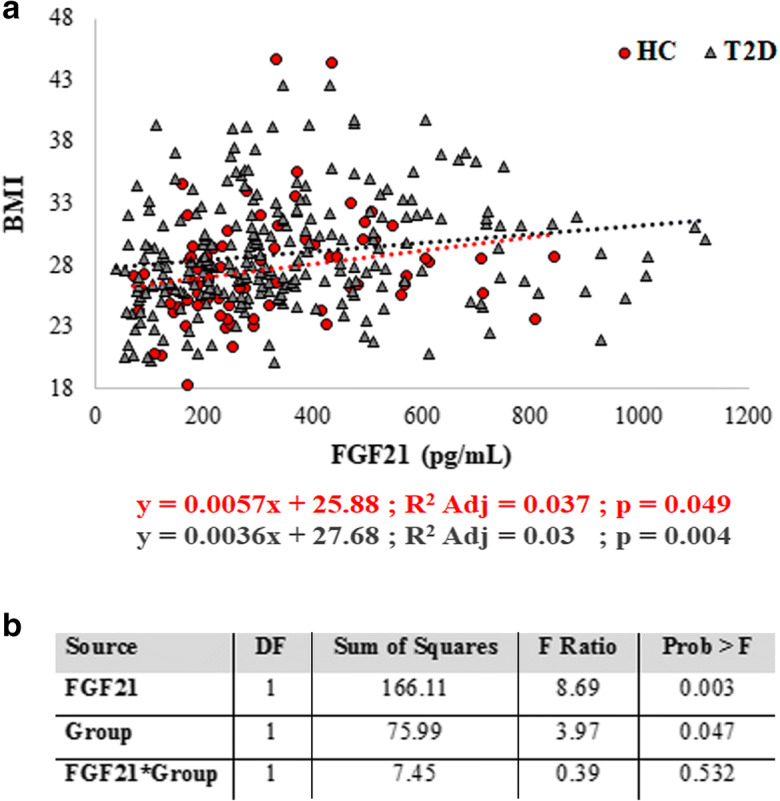
Regression analysis of FGF21 plasma levels with BMI. **a** Linear regression analysis of two different groups: healthy controls (HC) and T2D patients. **b** Comparison between the two regression curves. DF, degrees of freedom; Group: effect of the *i*th group (HC, T2D)

As far as the levels of FGF21 in T2D patients, our results are in apparent contrast with literature data showing that T2D patients are characterized by higher levels of FGF21 as compared to controls [42–44]. However, since FGF21 is influenced by BMI [44], and BMI is often higher in T2D patients than control subjects [45], we have hypothesized that the relationship between FGF21 and T2D can be mediated by BMI. To confirm this idea, we first divided HC and T2D patients in three subgroups according to their BMI (normal weight, overweight, and obese) and we observed that the levels of FGF21 were similar between HC and T2D in each BMI subgroup, while FGF21 levels were significantly higher in overweight and obese subjects with respect to normal weight group in both HC and T2D (Table 2). To further confirm these data, we then performed a regression analysis. A positive association between BMI and FGF21 concentrations is present; however, the two regression lines referred to as HC and T2D are not significantly different, confirming that FGF21 is influenced by BMI but not T2D (Fig. 2).

**Table 2:** Circulating levels of FGF21 in healthy controls (HC) and T2D patients subdivided by BMI. The comparison is shown between HC and T2D within the same BMI subgroup (*p* values reported on the right, Mann-Whitney test) and among the different BMI subgroups (*p* values reported below, Kruskal-Wallis test). Data are expressed as mean values ± standard error (SE). Significance level of *p* value is < 0.05. n.s., not significant

Mean ± SE	Normal weight (BMI range: 18.5 to 24.99)		
	HC (*n*° = 22)	T2D (*n*° = 40)	*p* value
BMI	23.16 (± 0.35)	23.16 (± 0.35)	
FGF21 (pg/mL)	258.32 (± 35.50)	286.59 (± 33.88)	n.s.
Mean ± SE	Overweight (BMI range: 25 to 29.99)		
	HC (*n*° = 49)	T2D (*n*° = 123)	*p* value
BMI	27.41 (± 0.20)	27.27 (± 0.12)	
FGF21 (pg/mL)	349.20 (± 40.66)	352.23 (± 28.35)	n.s.
Mean ± SE	Obese (BMI > 30)		
	HC (*n*° = 21)	T2D (*n*° = 92)	*p* value
BMI	33.45 (± 0.89)	33.71 (± 0.32)	
FGF21 (pg/mL)	377.72 (± 29.12)	412.95 (± 22.42)	n.s.
	*p* value *0.03*	*p* value *<0.001*

### Plasma levels of mitokines increase with age

**Fig. 3:**
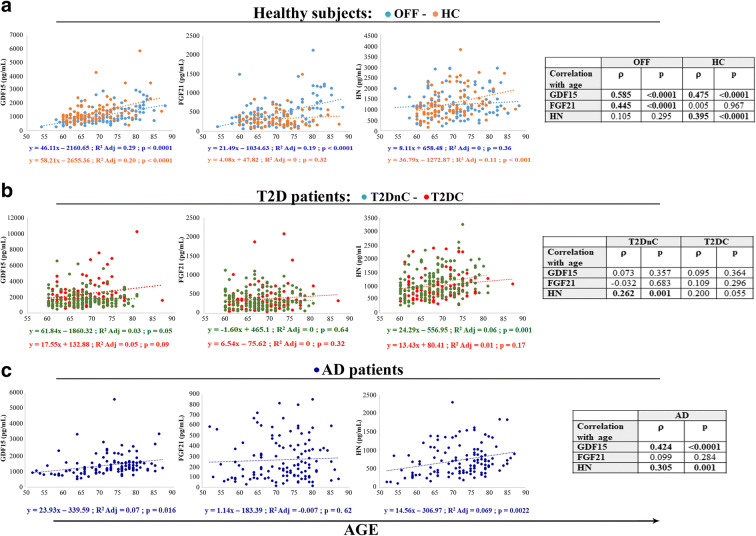
Regression analysis of GDF15, FGF21, and HN plasma levels with age. Linear regression and Spearman rank correlation analysis (ρ) between age and GDF15, FGF21, and HN in centenarians’ offspring (OFF) and healthy controls (HC) (**a**), T2D patients without complications (T2DnC) and T2D patients with complications (T2DC) (**b**), and Alzheimer’s disease patients (AD) (**c**)

We have previously reported that mitokine plasma levels increase with age in healthy people, from young to centenarians, and are correlated with worst hematochemical parameters, including lipid profile [5]. We then sought to check whether the age-related increase is confirmed in a narrower age range. Moreover, since in that previous study the participants were healthy subjects, we wondered whether similar changes of mitokines were present also in patients with T2D or AD. Plasma levels of GDF15, FGF21, and HN were all positively and significantly correlated with age in healthy subjects (data not shown), confirming previous results [5]. At variance, when looking at patients (T2D and AD pooled together), HN resulted strongly associated with age, as well as GDF15, although more weakly, but not FGF21 (data not shown). However, when we performed the regression analysis considering the subjects divided by groups (HC, OFF, T2DnC, T2DC, AD), the correlation with age was not always confirmed. GDF15 plasma levels were associated with age in OFF and HC, as well as in AD patients (Fig. 3a, c), but not in T2D patients (Fig. 3b). FGF21 plasma levels resulted associated with age only in OFF (Fig. 3a). HN plasma levels resulted associated with age in HC, T2DnC, and AD patients (Fig. 3a–c). Interestingly, no mitokine resulted associated with age in T2DC patients (Fig. 3b). This could be due to the excess fractionation into small groups, or to the presence of a disease that can overwhelm the effect of age, as already observed for the levels of circulating miR-146a in T2D patients [39].

### Mitokines in T2D patients

**Fig. 4:**
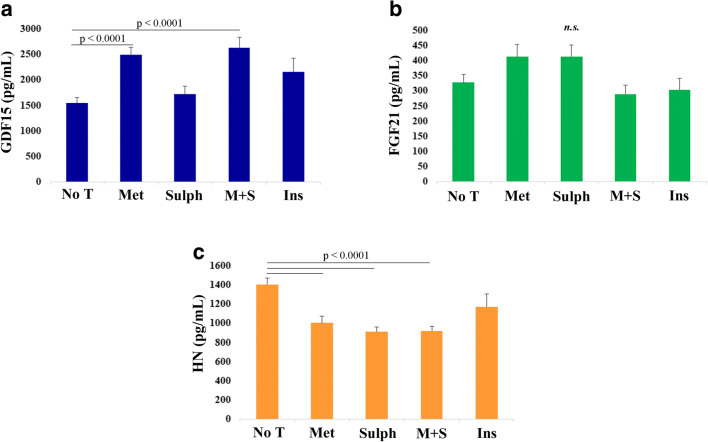
Plasma levels of GDF15, FGF21 and HN in T2D patients with different antidiabetic treatments. Comparison of circulating plasma levels of GDF15 (**a**), FGF21 (**b**), and HN (**c**) in T2D patients undergoing the following treatments: no treatment (No T), metformin (Met), sulphonylureas (Sulph), combination of metformin and sulphonylureas (M + S), and insulin (Ins). Data are expressed as mean ± SE. *p* values were determined by Kruskal-Wallis test with Bonferroni correction

After adjusting for age and gender, mitokines in T2D patients were analyzed in relation to the type of treatment, i.e., no treatment (NoT), metformin (Met), sulphonylureas (Sulph), combination of metformin and sulphonylureas (M + S), and insulin (Ins). The circulating levels of GDF15 were significantly higher in patients treated with Met (Fig. 4a) with respect to NoT patients or treated with Sulph or Ins. This result is in agreement with literature data indicating that GDF15 mediates the positive effects of metformin [28, 46]. Conversely, the levels of HN are higher in NoT patients in comparison to Met, Sulph, and M + S patients. No difference was found between NoT and Ins patients (Fig. 4c). No significant difference was observed for FGF21 (Fig. 4b).

Moreover, T2D patients often present abnormalities in lipid profile and high risk to develop cardiovascular diseases [47, 48] and are consequently treated with lipid lowering and antihypertensive therapies. Therefore, we evaluated the possible impact that an altered lipid profile or lipid lowering and antihypertensive therapies could have on mitokine levels. We first subdivided T2D patients on the basis of the triglycerides/HDL (TG/HDL) ratio, known to be associated to insulin resistance and cardiovascular disease in T2D [49, 50]. We found that patients with high TG/HDL ratio have higher level of GDF15 and FGF21 (Supplementary Table 1). At variance, the presence of lipid lowering therapy, antihypertensive therapy, or both, does not impact on mitokines levels (data not shown).

As far as T2D complications, the presence of a complication resulted often associated with a higher level of GDF15 (Table 3), also after adjusting for age, gender, and treatment. This result is in agreement with previous literature data [27, 51]. FGF21 resulted more elevated in presence of nephropathy or cardiac ischemia. Interestingly, for HN the situation was less linear: higher HN levels were found in presence of chronic kidney disease, while lower levels were found in presence of retinopathy and, in general, of at least one complication (Table 3). Moreover, only the plasma levels of GDF15 were significantly correlated with the number of complications (Spearman rank correlation coefficient and *p* value: rho = 0.265, *p* < 0.001).

**Table 3:** Circulating mitokines levels in T2D patients in relation to the different T2D-related complications. CAD, coronary artery disease; CKD, chronic kidney disease; PAD, peripheral artery disease; MACE, major adverse cardiovascular events. Variables are expressed as mean (standard error). *P* value derived from post-hoc tests with Bonferroni corrections after analysis of covariance (ANCOVA). Age, gender, and glucose-lowering treatment are considered as covariates

Complication	*N* (%)	GDF15 pg/mL (± SE)			FGF21 pg/mL (± SE)			HN pg/mL (± SE)		
		Absent	Present	*p*	Absent	Present	*p*	Absent	Present	*p*
Neuropathy	54 (21.2)	1877.3 (88.6)	2608.0 (197.4)	0.001	362.1 (19.4)	376.1 (43.3)	0.767	1079.7 (35.3)	1088.4 (78.6)	0.920
CKD	24 (9.4)	1931.1 (81.6)	2848.1 (257.1)	0.001	349.5 (17.5)	521.7 (55.2)	*0.003*	1065.3 (32.5)	1161.9 (102.4)	0.370
Retinopathy	74 (29.0)	1841.3 (92.2)	2446.2 (145.5)	0.001	351.9 (20.2)	387.9 (31.8)	0.342	1119.3 (36.4)	951.3 (57.4)	*0.014*
PAD	10 (3.9)	2023.0 (81.2)	1961.9 (410.1)	0.884	365.1 (17.3)	362.2 (87.2)	0.973	1071.5 (31.6)	1126.6 (159.6)	0.735
CAD	25 (9.8)	1965.5 (82.8)	2675.8 (268.7)	0.012	350.4 (17.3)	562.3 (56.2)	< 0.001	1071.3 (32.7)	1109.6 (106.0)	0.730
MACE	29 (11.4)	1976.4 (83.6)	2609.2 (260.8)	0.022	359.6 (18.1)	424.0 (56.4)	0.278	1074.1 (33.0)	1078.4 (103.0)	0.968
At least one complication	101 (39.6)	1810.2 (101.0)	2357.1 (125.5)	0.001	356.2 (22.0)	383.8 (27.3)	0.434	1124.5 (39.9)	995.4 (49.6)	0.044

The significance level of *p* value is < 0.05 and written in italic

Since the levels of the three mitokines were higher in T2D patients with at least one complication, a binary regression analysis was performed to estimate the odds ratio (OR) and 95% confidence interval (CI) of mitokines for predicting the presence of diabetic complications. In addition to mitokines, age, gender, and serum HbA1c were included in this model as covariates, as it is well known that glycemic control is closely associated with the onset of complications [52, 53]. Binary logistic analysis showed that among mitokines, only GDF15 was independently associated with diabetic complications (Table 4). Remarkably, the association remained significant despite rigorous adjustment for conventional disease-related variables.

**Table 4:** Binary logistic regression analyses of variables contributing to diabetic complications (M = male). The logistic regression model is statistically significant (*χ*2(4) = 90.838, *p* < 0.0001), explains 33.4% of the total variance (Nagelkerke *R*^2^) and correctly classifies 76.0% of cases. The Odds ratio of mitokines refers to the increased probability to have a complication per every 100 pg/mL of mitokines. *P* value derived from post-hoc tests with Bonferroni corrections after analysis of covariance (ANCOVA). Age and gender are considered as covariates

Parameters	B	SE	Wald	Odds ratio	95% CI for Odds ratio	p value
Gender (M)	0.742	0.285	6.803	2.101	1.203–3.670	0.009
Age	0.057	0.026	4.928	1.059	1.007–1.114	0.026
HbA1c	0.923	0.141	42.760	2.518	1.909–3.321	< 0.001
GDF15	0.027	0.012	4.639	1.027	1.002–1.053	0.031
FGF21	− 0.026	0.052	0.256	0.974	0.880–1.079	0.613
HN	− 0.056	0.030	3.545	0.945	0.891–1.002	0.060

The significance level of *p* value is < 0.05 and written in italic

**Fig. 5:**
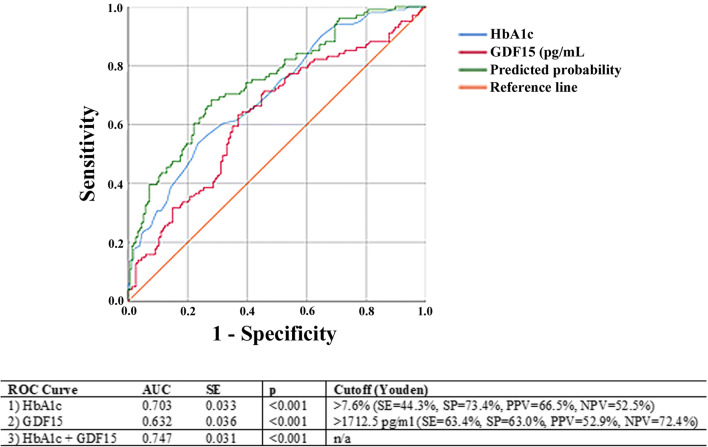
Receiver operating characteristic (ROC) curves for GDF15 and hemoglobin A1c (HbA1c) in T2D patients with complications. Comparison of ROC analysis of GDF15, HbA1c, and GDF15 + HbA1c. GDF15 and HbA1c together gave a value of area under the ROC curve (AUC) larger than those of the two parameters analyzed separately

To evaluate the discriminative ability of GDF15 for the presence of complications, we calculated the receiver operating characteristic curve (ROC) analysis and compared with that of HbA1c (Fig. 5). The AUCs for GDF15 and HbA1c were 0.632 (*p* < 0.001) and 0.703 (*p* < 0.001), respectively. When combined together, GDF15 and HbA1c gave an AUC value of 0.747 (*p* < 0.001), which is significantly larger than those for the two separated parameters (HbA1c + GDF15 versus HbA1c, *p* = 0.013; HbA1c + GDF15 versus GDF15, *p* = 0.003) (Fig. 5). Taken together, these results indicated that GDF15 could significantly improve the reliability of HbA1c in the assessment of glycemic control and in the diagnosis of T2D complications.

**Fig. 6:**
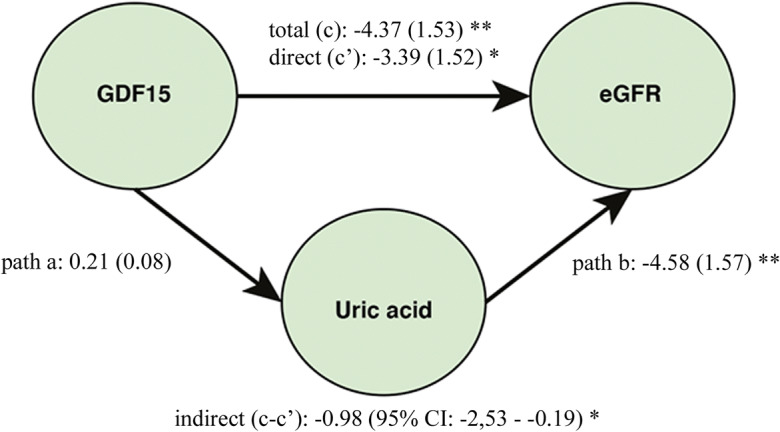
Conceptual framework of the mediation analysis. Direct acyclic graph showing the association between GDF15 and estimated glomerular filtration rate (eGFR), where uric acid was considered as mediator. *p < 0.05; **p < 0.01 for standardized bootstrapped (10,000 samples) total, direct and indirect effect size. In brackets, the standard errors for total and direct effects and the 95% CI for indirect effect are reported. The mediation procedure is described in the “Material and methods” section

Recent data suggest that GDF15 can be a risk predictor of kidney function decline [24]. Besides, we have observed that high levels of mitokines correlated with nephropathy/chronic kidney disease (Table 3), as well as with levels of creatinine and estimated glomerular filtration rate (eGFR) (Table 5). We therefore tested the hypothesis that the effect of mitokines on kidney function could be mediated by other variables. The mediation analysis was performed testing several variables known to be linked to kidney disease, including C-reactive protein, IL-6, uric acid, azotemia, triglycerides, ApoA1, ApoB, and HbA1c, and that resulted associated with mitokines, (data not shown). Results indicate that FGF21 and HN have an independent effect on eGFR, while the 22% of the effect of GDF15 on eGFR was mediated by uric acid (− 0.98/−4.37 = 22.4%) (Fig. 6).

**Table 5:** Spearman rank correlation coefficient and *p* values for the correlations between circulating mitokines and renal function, expressed as serum creatinine and eGFR, in T2D patients

Correlations	Creatinine		eGFR	
	*ρ*	*p value*	*ρ*	*p value*
GDF15	0.461	< 0.001	− 0.301	< 0.001
HN	0.323	< 0.001	− 0.318	< 0.001
FGF21	0.209	0.001	− 0.163	0.009

### Mitokines and ApoE genotype

**Fig. 7:**
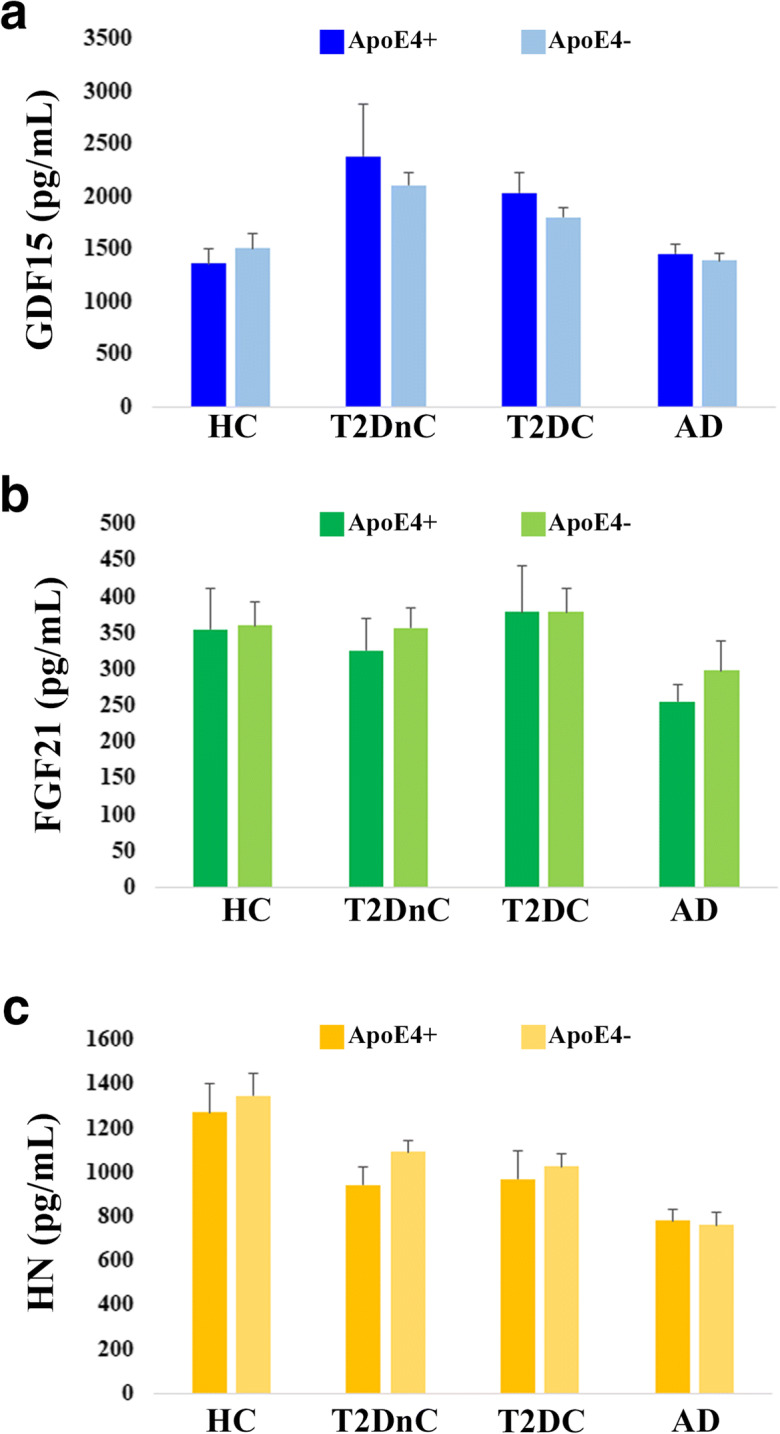
Plasma levels of GDF15, FGF21, and HN in ApoE4 carriers and non-carriers subjects. Circulating plasma levels of GDF15 (**a**), FGF21 (**b**), and HN (**c**) in healthy controls (HC), T2D patients without complications (T2DnC) and T2D patients with complications (T2DC), and Alzheimer’s disease patients (AD) divided by ApoE4 carriers and non-carriers subgroups. Data are expressed as mean ± SE

As described above, AD but not T2D patients show lower plasma levels of HN as compared to HC and OFF, while a specular situation is found for GDF15: T2D but not AD patients have higher levels as compared to HC and OFF. Finally, a difference exists between T2D and AD patients as far as FGF21 (Fig. 1). This suggests that despite a tight metabolic connection between AD and T2D [9, 10], a clear difference exists as far as mitokine expression pattern in these two pathologic conditions. ApoE4 allelic variant is a universally recognized risk for sporadic AD and affects mitochondrial function, energy, and lipid metabolism in AD patients [54–56]. So far, no data are available as far as the association between ApoE4 and mitokines. We therefore investigated the possible association between ApoE4 allele and the levels of mitokines. ApoE genotypes for the majority of subjects (AD and T2D patients, part of HC) were available and are shown in Supplementary Table 2. When looking at all subjects pooled together (HC, T2DnC, T2DC, and AD), only for HN we found a slight difference between ApoE4 carriers and non-carriers. In particular, HN levels were lower in ApoE4+ subjects compared to ApoE4− ones (*p* = 0.04, data not shown). However, when we performed the same analysis considering the subjects divided by groups, no difference in the levels of the three mitokines was observed between ApoE4 carriers and non-carriers (Fig. 7).

## Discussion

The mainstream interpretation of the biological role of mitokines is that they may help tissues, organs, and eventually the whole organisms in coping with stresses by mediating metabolic adaptation in response to an energy crisis produced by mitochondrial dysfunction [5, 6, 57]. However, many findings indicate that high circulating levels of GDF15, FGF21, and HN are associated not only with beneficial response to mitochondrial dysfunction but also with aging and several age-related diseases to the point that they are often considered useful diagnostic markers [6, 58–60]. In agreement with this tenet, in our previous studies, we have shown that these mitokines increase with age and are particularly elevated in centenarians, as well as in subjects that presented worse values of biochemical parameters, including insulin resistance (HOMA-IR), lipid profile, and inflammation [5, 24]. However, it is not clear whether different age-associated diseases characterized by mitochondrial dysfunction display similar patterns of mitokine expression. To this regard, we focused on two very common age-related diseases, T2D and AD, for which several studies suggested that insulin resistance and mitochondrial dysfunction could be the common denominators [61, 62]. T2D is a devastating disease, causing excessive rates of cardiovascular disease, renal disease, eye diseases, and many neurological problems. AD itself has been recently proposed as an additional complication of T2D [8, 63]. This idea is supported by evidence indicating that the decrease of glucose metabolism caused by insulin resistance results in stress at mitochondrial level, leading to apoptosis of neurons and neuroinflammation [61, 62].

In the present study, we compared the plasma levels of GDF15, FGF21, and HN in T2D and AD patients as compared to healthy subjects, including centenarians’ offspring (OFF) who are reported to be in a better health status as compared to age-matched peers [35, 36], and we observed a differential expression pattern of these mitokines at circulating level. Based on previous studies [5, 6] and literature data (in particular for GDF15), we were expecting that patients would display higher levels of mitokines with respect to healthy controls. In contrast with these expectations, the results showed a more complex situation. In particular, circulating GDF15 levels were higher in T2D patients but not in AD ones, FGF21 levels were elevated in OFF and lower in AD but not in T2D patients, and HN levels were lower in both T2D and AD patients, particularly in the latter. Moreover, we observed a significant association of all three mitokines with age only in healthy subjects, while in T2D and AD patients this association appears to be not always present. Consistently, mitokines correlated with each other only in OFF and HC, but not in patients (data not shown). These results suggest that, when considering healthy subjects, age is a determinant of mitokine increase, while when considering subjects of similar age range but different health status, the level of the three mitokines, and in particular GDF15 and FGF21, may greatly vary.

Regarding T2D, all the three mitokines showed interesting associations with the presence of complications and they were related to worsening eGFR. In particular, while confirming literature data indicating that GDF15 increases in T2D [27, 64], our results indicate that GDF15 improves the ability of the conventional marker HbA1c in diagnosing patients with complicated T2D. To this regard, the identification of novel biomarkers capable of predicting the development of complications and the decline of renal function in patients with T2D is a timely issue.

Conversely, for FGF21, we did not observe any association with T2D. As mentioned in the “Results” section, literature data indicate that the levels of FGF21 are significantly higher in patients with T2D with respect to healthy controls [42–44]. However, FGF21 is strongly related to obesity [17], and we have observed that, when stratified for BMI, T2D patients and healthy controls have the same level of FGF21, suggesting that the reported association of FGF21 with T2D is likely mediated by BMI. In any case, the association of FGF21 with BMI and obesity appears to be paradoxical, since it has been demonstrated that FGF21 overexpression or administration prevents diet-induced obesity and insulin resistance [65, 66]. It is possible that the secretion of FGF21 in overweight people is an adaptive attempt trying to control weight gain [67].

As far HN and T2D, we did not observe a strong association between them; however, the levels of HN tended to be lower in T2D patients compared to HC, although not significantly after Bonferroni correction. The possible role of HN in T2D is still unclear, and to date, there are still few studies on HN in diabetes. In particular, Voigt and Jelinek showed that the plasma levels of HN are lower in prediabetic patients with impaired fasting glucose compared to a control group [68], and similarly, Ramanjaneya and co-workers demonstrated that serum HN concentration was lower in T2D and negatively correlated with HbA1c and glucose [69]. In agreement, in our study, we also found a negative correlation of HN with HbA1c (spearman’s rho = −0.305, *p* < 0.0001) and glycemia (spearman’s rho = −0.291, *p* < 0.0001), suggesting that HN could be involved in the maintenance of insulin sensitivity. Taken together, these data suggest that GDF15 and HN are involved in the response to diabetic stress with an opposite regulation, while FGF21 appears to be affected by BMI but not T2D.

Concerning AD, limited data are available on the association between GDF-15 and neurodegenerative diseases. In our study, the levels of GDF15 in AD patients were similar to those of healthy controls. Some studies reported that higher levels of GDF-15 (plasma, serum, or cerebrospinal fluid) are associated with cognitive impairment and dementia, as well as with decreased gray matter volumes and white matter integrity. All these data suggest that GDF15 could be a possible biomarker for neurodegenerative diseases [70–72]. However, none of these studies described the levels of GDF15 for AD patients, but rather for Parkinson’s disease or Lewy Body Dementia. Thus, GDF15 plasma levels may be associated to some neurodegenerative diseases, but not to AD.

As far as FGF21 in AD patients, it has been reported that higher levels of FGF21 have beneficial effects in several pathologies, including neurodegenerative diseases, although the biological function of FGF21 on AD is still largely unclear. To this regard, an in vitro and in vivo study reported that FGF21 attenuated the negative effects of amyloid β-peptide 25–35 on neuronal apoptosis, tau hyperphosphorylation, and oxidative stress in AD-like pathologies [73]. In agreement with these data, we found that FGF21 circulating levels in AD are lower with respect to the other groups, in particular to OFF. Interestingly, we recently reported that post-menopausal women suffering by chronic insomnia, a condition known to be a risk factor for the development of AD [74], display lower levels of FGF21 as compared with age-matched women without sleep disorders [75]. This result further supports the idea that FGF21 plays important roles in neurophysiology.

As far as HN in AD, our results confirm literature data indicating that HN decreases in AD [76–78]. In our samples, HN was, in fact, lower in AD patients with respect to healthy controls. Interestingly, although several studies showed a decrease of HN with aging, our results showed a strong positive correlation of HN with age in AD patients. This suggests that the mechanisms that impinge upon HN production are different and independent in AD and aging. Moreover, we did not find any difference between OFF and HC, at variance with a recently published study [79]. To this regard, it is to note that the authors of this study reported very low levels of HN for both OFF and HC (around 500 pg/ml and 200 pg/ml, respectively) that are much lower than expected according to previous studies (1200 pg/ml or higher) [32, 74, 80, 81]. Moreover, they used an in-house kit for the detection of HN, while we used a commercially available one (see the “Materials and methods” section), so the two studies are likely not comparable. Further investigations are needed to clarify this point.

As a whole, our results suggest that GDF15, FGF21, and HN may act synergistically only during physiological aging in the absence of overt diseases. Conversely, when a disease occurs, their expression is modulated differently, even though the considered diseases share a common ground of mitochondrial impairment, like T2D and AD. More studies are needed to clarify the mechanisms underlying this differential modulation.

## Supplementary Information

ESM 1 (DOCX 17 kb)
