# Supplementary material for: Disease-specific plasma levels of mitokines FGF21, GDF15, and Humanin in type II diabetes and Alzheimer’s disease in comparison with healthy aging
Source: GeroScience. 2020 Oct 31;43(2):985–1001. doi: 10.1007/s11357-020-00287-w (PMC8110619; doi:10.1007/s11357-020-00287-w)
Supplement: Supplementary file 1 — (DOCX 17 kb) [file 11357_2020_287_MOESM1_ESM.docx]

**Supplementary Tables**

**Supplementary Table 1**

Comparison between T2D patients with low or high Triglycerides/HDL (TG/HDL) ratio. The median score = 2.2 was used as a cut off to divide T2D patients in high TG/HDL and low TG/HDL groups.

| **Values**  **(mean ± SD)** | **LOW TG/HDL group,**  **n = 127** | **HIGH TG/HDL group,**  **n = 128** | **p values** |
| --- | --- | --- | --- |
| Age | 68.65 ± 5.58 | 68.91 ± 5.03 | n.s. |
| BMI | 27.77 ± 4.56 | 30.07 ±4.03 | < 0.0001 |
| TG/HDL (Range) | 1.38 ± 0.5 (0.49-2.17) | 4.57 ± 3.3 (2.25-23.53) | < 0.0001 |
| GDF15 (pg/mL) | 1845.42 ± 861.84 | 2193.45 ± 1556.56 | 0.028 |
| FGF21 (pg/mL) | 286.76 ± 192.45 | 440.33 ± 312.08 | < 0.0001 |
| HN (pg/mL) | 1027.55 ± 453.9 | 1115.69 ± 551.4 | n.s. |

**Supplementary Table 2**

APOE genotype in AD and T2D patients and healthy controls (HC).

| **ApoE**  **genotype** | **AD**  **n (%)** | **T2DnC**  **n (%)** | **T2DC**  **n (%)** | **HC**  **n (%)** |
| --- | --- | --- | --- | --- |
| E2/E2 | 0 (0%) | 0 (0%) | 1 (1.12%) | 0 (0%) |
| E2/E3 | 7 (5.83%) | 14 (10.38%) | 15 (16.86%) | 6 (9.52%) |
| E2/E4 | 2 (1.67%) | 3 (2.22%) | 1 (1.12%) | 0 (0%) |
| E3/E3 | 53 (44.17%) | 100 (74.07%) | 60 (67.41%) | 47 (74.61%) |
| E3/E4 | 44 (36.67) | 16 (11.85%) | 12 (13.49%) | 8 (12.7%) |
| E4/E4 | 14 (11.66%) | 2 (1.48%) | 0 (0%) | 2 (3.17%) |
